# Adopting the principles and practices of learning health systems in universities and colleges: recommendations for delivering actionable data to improve student mental health

**DOI:** 10.1080/28324765.2023.2301339

**Published:** 2024-01-22

**Authors:** Emma Broglia, Michael Barkham

**Affiliations:** a Department of Psychology, University of Sheffield, Sheffield, UK; b Clinical and Applied Psychology Unit, Department of Psychology, University of Sheffield, Sheffield, UK

**Keywords:** Mental health, students, service user involvement, outcome measures, learning health systems

## Abstract

Despite efforts to address the growing severity and complexity of student mental health issues, challenges persist in data quality and application, thereby hindering progress. This article is built upon three decades of cumulative expertise from the authors, complemented by insights gathered through the extensive observations of students and practitioners within the realm of student mental health. Drawing from both robust evidence and hands-on experience, it presents evidence-based recommendations aimed at enhancing data capture and utilization in alignment with the principles and methodologies of learning health systems. The approach is based on four interconnected themes: (1) Learning from global insights on university students’ mental health; (2) Measuring, monitoring, and managing data; (3) Involving student and stakeholder perspectives; and (4) Mapping transitions, access, and systems for student mental health services. These themes encompass both the individual student journey and the broader organizational layers of universities and colleges. Ten recommendations are made for supporting universities and colleges to move towards adopting the principles of learning health systems to support students’ mental health.

In recent years, considerable attention across a range of media has focused on the state of student mental health. While various strategies have been adopted to capture and respond to students’ needs, global trends indicate increasing severity and complexity (Lipson et al., 2019). Diverse research methods have also been employed to address these needs, such as using large practice-based datasets, systematic reviews and meta-analyses, national and international surveys, and longitudinal data capture (e.g., Hazell et al., 2020; Paton et al., 2023). The resulting evidence has provided new insights into student mental health trends and strengthened the evidence base (Campbell et al., 2022). Nevertheless, challenges persist in the quality of data and its utilization, impeding progress in the field, and impeding universities and colleges not only in collecting actionable data but also in being the beneficiaries of the lessons learnt from the data. Moving in such a direction relies increasingly on utilizing digital technology, connecting systems and infrastructure, and leveraging datasets to enhance in-house (i.e., within the institution) support services and thereby improve student mental health outcomes. This article aims to provide a step toward responding to these data challenges by synthesising evidence-based recommendations for capturing and reporting mental health trends in university and college students.

Our approach draws from over 30 years of collective experience in the realm of student mental health, collaborating with students and practitioners at various levels, and from frontline staff to service leads and directors. We specifically reference psychometrics, research and evaluation, policy, and data informatics to offer initial insights into key concepts required to move towards data-informed student mental health systems. Crucially, our approach draws on a history of working with students, service users, practitioners, heads of services, and professional organizations from the university and college counseling sector to understand the unique challenges they face in their roles in supporting student mental health and wellbeing. In particular, we draw on our experience from the UK Student Counselling Outcomes Research and Evaluation (SCORE) practice-research consortium (see https://score-consortium.sites.sheffield.ac.uk/).

On the basis of this collective experience, we have been drawn to the concept of learning health systems, a concept first outlined by the Institute of Medicine (IOM, 2001) in which the generation of evidence (i.e., data) is a “by-product of care delivery” and the application of that evidence is “to support continuous improvement, evidence-based care delivery, and population management” (Guise et al., 2018, p. 2237). In effect, evidence generation is paired with evidence application such that “evidence is both generated and applied as a natural product of the care process” (Ramsberg & Platt, 2017). A key principle of learning health systems is that the process is a continuous cycle where new knowledge, new data, and new perspectives lead to practice changes which in turn provide new knowledge and so the cycle continues. Currently, at least in the UK, universities do not have a legal duty of care to students in the same way as they do for students aged under 18 and also to their staff, but it has become an issue due to the tragic occurrence of student suicides. We argue that universities and colleges can position themselves to adopt the principles and practices of learning health systems to maximize the natural process of care towards students and their mental health.

In line with this approach, we have identified four themes that are summarized in Figure 1 and act to organize key issues and considerations: (1) Learning from global insights on university and college students’ mental health; (2) Measuring, monitoring, and managing health data; (3) Involving students and stakeholder perspectives; and (4) Mapping transitions, access, and systems for student mental health services. These themes are building blocks-not a comprehensive template-that acknowledge a cyclical sequence moving from the collection of research evidence and local data, through participation with stakeholders, and towards changes in practice that are consistent with the principles of learning health systems (Agency for Healthcare Research and Quality, 2019). Central to these principles is the practice of embedding routinely collected outcome measures at each session, providing the infrastructure and resources for services to access and utilize their data, empowering a culture of change and commitment from the organization to learn from the data, as well as an interface with learning from broader, more global evidence.

**Figure 1.**
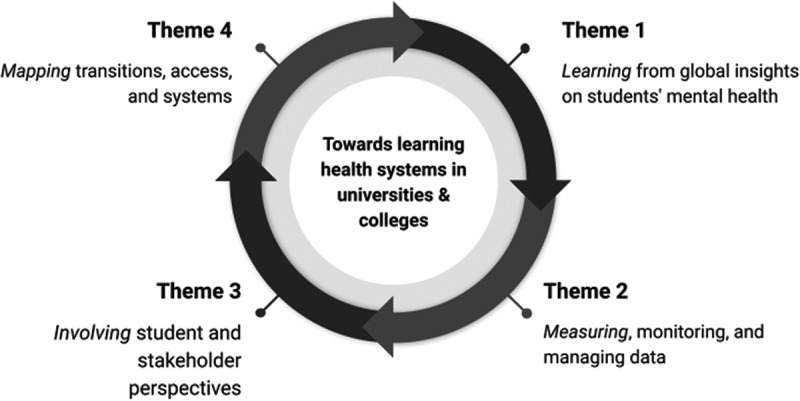
Framework of themes that underpin universities/colleges adopting principles and practices of learning health systems.

Our approach starts by adopting a population approach to identify global insights into student mental health trends and explore potential enablers to inform local clinical groups within the student population. Through this approach, we acknowledge the value of learning from the broader, global literature and incorporating studies of universities/colleges across multiple countries that showcase good practice. Progressing through the themes, we focus on the generation of primary data from individual students and their evolving engagement with mental health support systems over time. As such, natural and routine data generation underpins the principle of a learning health system. Combined with the perspectives and involvement of students themselves, the impact leads to mapping the transitions to universities/colleges, improving access, and developing more effective and efficient mental health systems for students.

## Theme 1: learning from global insights on university students’ mental health

The first theme adopts a global public health perspective and explores the global literature as a potential source for information about courses of action that contribute knowledge. Understanding the knowledge and insights from a public or population level has the potential to identify overarching enablers that can direct research and interventions for local clinical populations (i.e., treatment-seeking students and service users). This externally-focused theme addresses a crucial axiom in terms of the principles and procedures of a learning health system.

### Global perspectives

A global perspective on university student mental health represents research from national, international, or multi-country studies that have explored trends from large or longitudinal student samples either directly from datasets or via meta-analyses (e.g., Lipson et al., 2019; Paton et al., 2023). These studies provide insights into the prominent mental health issues among students, potential risk factors that contribute to poor mental health, and the strategies needed to improve student outcomes. Exploring global perspectives helps identify students’ needs and direct service development and research. This broader perspective also allows us to identify common challenges and successful approaches that can be adapted to different contexts.

Numerous studies have investigated mental health trends among university and college students, consistently revealing a rise in mental health conditions over time. Noteworthy research, such as Duffy et al. (2019) and Oswalt et al. (2020), utilized longitudinal data on self-report student mental health symptomatology including the National College Health Assessment (*n* = 610,543), Healthy Minds Study (*n* = 177,692), and American College Health Association-National College Health Assessment (*n* = 454,029). Analyses of these datasets have shown increasing trends for depression, anxiety, panic, self-injury, suicidal ideation, and suicidal attempts, with several incidences doubling over time.

Similar trends of deteriorating mental health have been observed in different countries and among specific student groups. For instance, in Norway, university students, especially women, experienced a 16.5% increase in psychological distress from 2010 to 2018 (Knapstad et al., 2021). In the UK, reports have indicated heightened anxiety, depression, loneliness, and a reduced sense of belonging among students (Campbell et al., 2022). Additionally, student-athletes, transfer students, and those facing financial difficulties have experienced higher levels of anxiety, depression, academic stress, and suicidal ideation (Bantjes et al., 2022; Cheung et al., 2020; Karyotaki et al., 2020; Rolland et al., 2022). Overseas students and home students from ethnic minority backgrounds further report unique challenges that affect their mental health including cultural and language barriers, racism, and stigma (Alang, 2019; Cao et al., 2021).

### World Health Organization (WHO) World Mental Health International College Student (WMH-ICS) initiative: an exemplar

One noteworthy and ongoing program of work is the World Health Organization (WHO) World Mental Health International College Student (WMH-ICS) initiative, which aims to address mental health problems among college students worldwide. This initiative generates a large, longitudinal epidemiological dataset to gain insights into common mental disorders and emotional problems in university and college students (Cuijpers et al., 2019a, 2019b). The WMH-ICS’s priority areas include generating epidemiological data, implementing digital approaches, disseminating evidence-based interventions, and facilitating regular evaluation through annual surveys. Its collaborations with 18 countries demonstrate its global commitment to addressing mental health needs, providing effective interventions for students, and improving accessibility to mental health services.

Recent findings from a cross-national sample of the WMH-ICS data revealed that only 24.6% (*n* = 3,440) of students were willing to approach professional services for emotional problems (Ebert et al., 2019). Common reasons for help avoidance included preferring to handle issues alone (56.4%) and seeking support from friends or relatives (48.0%). Similar reluctant help-seeking has been reported in the UK with further concerns about the time commitment for using services and experiencing self-stigma (Broglia et al., 2021). These findings highlight substantial hesitation among students in seeking mental health support and indicate the need for meaningful co-development with students to personalise communication and promote appropriate early engagement.

A second noteworthy exemplar of combining longitudinal clinical data from university and college counseling centers is the Center for Collegiate Mental Health (CCMH). CCMH connects approximately 750 college counseling centers that have adopted a single student-specific measure-the Counseling Center Assessment of Psychological Symptoms (CCAPS) measure as well as standardized data forms for presenting issues and demographics. Since its establishment in 2004, CCMH has gathered data from 1.5 million clients and generated over 80 publications and 15 annual reports. The latest report presents insights from 180 centers, 190,907 students, and 4,688 clinicians across 1,287,775 appointments. Recent analyses highlight student risk and protective factors for academic success as well as a noticeable increase in reported traumatic events, social anxiety, and marginal rises in thoughts of harming others (see CCMH Annual Report, 2022).

Recognising and addressing risk factors that contribute to students’ poor mental health is crucial in meeting their unique needs. Multiple studies have conducted systematic reviews and meta-analyses to identify such risk factors as well as specific student groups who are more susceptible to experiencing mental health challenges (e.g., Hazell et al., 2020; Pacheco et al., 2017). Early studies suggest that risk factors for poor mental health include race/ethnicity, religiosity, relationship status, living arrangements, and financial situation (Eisenberg et al., 2013). In a recent study, Sheldon et al. (2021) identified significant predictors of depression and suicide-related outcomes in undergraduates including current mental health problems, negative rumination, parent separation, parental depression, childhood adversity, sexual harassment, and financial difficulties.

Specific risk factors have also been identified in the UK, such as childhood trauma, LGBTQ identity, and autism (Campbell et al., 2022). At the same time, protective factors include having supportive social networks and feeling prepared for the university transition, while lack of engagement and low mental health literacy contribute to poor mental health. Factors specific to the education context have also been identified. For example, Limone and Toto (2022) found that study time, loneliness, and long-term stress predict anxiety and depression as well as childhood trauma, abuse, and neglect. Recognising and responding to these risk factors provide the potential to inform tailored communications, interventions, and service pathways for students.

## Theme 2: measuring, monitoring, and managing data

The second theme underscores the significance of systematically collecting and analyzing local data to improve mental health services, decisions, and interventions. This theme considers the importance of comparing embedded measures for shared assessments and comparability and the issues arising from implementing common metrics and standardized approaches in pursuit of improved data quality and reporting of university and college student mental health.

### Measurement: standardization vs. idiographic

Several strategic decisions about measurement are key and standardization is a good guiding principle. Standardization can be realized both in the procedures used (i.e., using the same measures) and also in terms of adopting nomothetic measures (i.e., invariant items). Employing measures that allow direct comparisons with other services and countries holds significant appeal and considerable merit. This approach can substantiate the practical aspects of data-sharing. However, standardization can result in the dominance of a single measure that becomes imposed and freezes the field whereby researchers, policymakers and service leads can be reluctant to migrate to a different measure for fear of losing comparisons with existing datasets. Lessons from psychological therapies have shown how a measure such as the Beck Depression Inventory (BDI) dominated the field before being overtaken by a free-to-use, briefer measure, namely the Patient Health Questionnaire-9 (PHQ-9), derived directly from the diagnostic nosology of the Diagnostic Statistical Manual. The tension arises in choosing between a diagnostically-based measure widely utilized in general psychological literature, offering valuable comparator data, and a focus on what may be more meaningful to students.

What is most meaningful to any given student might better be captured by a battery of brief measures or the adoption of idiographic measures. These may be more time-consuming to elicit but can be very insightful if chosen carefully. In effect, idiographic measures are *individualized* and they engage directly with a student in the construction of a measure -*their* measure. The idiographic approach provides a more flexible and individualized method to understanding client needs and progress. When idiographic measures have been implemented into adolescent mental health services they have improved collaboration between clients and counsellors and enabled adolescents to convey the seriousness of their concerns. Moreover, counselors described the approach as empowering clients by focusing on “what matters” to clients (Tollefsen et al., 2020).

The adoption of idiographic items is an interesting perspective in an increasingly digital world for a method that dates back to the 1960s (e.g., Shapiro, 1961, 1969). It also directly connects clients with measurement data in a way that nomothetic measures are not able to do. This connection is particularly beneficial to universities and colleges given the specific and unique pressures students face. These contextual factors might suggest that items within idiographic measures enable a more personalised approach centered on student experiences, which in turn, foster greater engagement. Jacob et al. (2023) has taken the idiographic approach one step further as a means to address some of the limitations of standardised measurements in psychotherapy by introducing the concept of idiographic patient reported outcome measures (I-PROMs). This approach combines I-PROMS with traditional measures to capture both individual progress and team-level changes to focus on client-specific needs over a pure symptomology approach. This focus shows promise for universities and colleges to bring focus on what matters to clients/students and to move away from a traditional medicalised model.

### Assessment and outcome

A further issue is to ensure there is a clear understanding of the difference but also the relationship between assessment and outcome. They are different processes and have different functions. It’s therefore logical to have different measures for each purpose. The widespread adoption of the PHQ-9 measure in mental health research and in psychological therapies has compounded the issue of assessment and outcome, making them become one and the same. There are advantages in having separate measures for different functions, and this can become important when considering using measures to monitor and feedback progress (see next sub-theme). Some pairings of measures provide a balance between comprehensive assessment instruments and briefer ongoing monitoring instruments: for example, the CCAPS-62 (Broglia et al., 2017; Locke et al., 2011; assessment) and CCAPS-34 (Locke et al., 2011; outcome monitoring); the CORE-OM (Evans et al., 2002; assessment) and CORE-10 (Barkham et al., 2013; outcome monitoring).

Our combined experience, involving the use of these measures in research and their application in therapy by practitioners, reveals that the decision-making process for clinic managers and service heads inevitably involves trade-offs. The CCAPS-62, widely embraced in counseling centers across the USA, offers a comprehensive assessment tailored for students, capturing mental health concerns and contextual experiences like academic distress. However, concerns about the length of measures have led practitioners to hesitate in adopting routine outcome monitoring through concerns about client burden (e.g., Barkham et al., 2023). Concurrently, the CORE-OM and its abbreviated form, the CORE-10, are prevalent in UK university and college counseling services. While not specifically designed for students, the CORE measures, particularly the CORE-10, have been commended for their brevity and sensitivity to change, vital for assessing improvement or deterioration in these short-term services (e.g., Mair, 2016).

The CCAPS measures have garnered substantial attention in the USA, implemented by numerous counseling centers along with standardized data forms covering presenting issues and demographics. Notably, the Center for Collegiate Mental Health (CCMH) has driven the adoption of standardized data forms that outline student service experiences, encompassing key categories like presenting issues, demographics, service pathways, and client feedback. This comprehensive approach, integrating routine outcome monitoring and standardized data, enabled CCMH to derive insightful perspectives on student mental health, resulting in impactful strategies and approaches.

However, in the UK, the adoption of CCAPS measures varies, presenting a persistent data challenge: services employ diverse measures and systems (e.g., Broglia et al., 2017). The disjointedness resulting from different measures and systems in the UK context impedes insights into student service user populations. Furthermore, combining diverse datasets in the UK compromises data quality due to the use of unstandardized surrounding data fields (Broglia et al., 2021). To alleviate these issues, implementing a standardized minimum dataset, akin to those used by CCMH and NHS, would prove advantageous for the UK and other countries facing similar data limitations. Addressing these challenges remains crucial in the UK, particularly as varied measures and systems create disjointed student journeys, potentially impeding future help-seeking. This issue is heightened in recent UK initiatives aiming to bridge university and NHS mental health services. Improved data linkage and aligned reporting standards are vital to facilitate effective partnerships (see Broglia et al., 2023).

While there are many popular outcome measures employed in the broader psychotherapy literature, one interesting measure that has also been adopted in university/college counseling centers is the Outcome Questionnaire-45 (OQ-45, Lambert et al., 1996). The OQ-45 aims to measure psychological distress and mental health status overtime with insights into clients’ functioning, relationships, and symptoms. Two particular studies in university/college settings standout that showcase the benefits of adopting outcome measures to facilitate nuanced approaches to understanding student mental health needs and outcomes. One example includes analysis of 6000 client records to identify predictors of student distress and noted particular concerns for students who demonstrate high levels of perfectionism or religiosity (Solomon et al., 2020). In a second study, outcome data from the OQ-45 were analysed to explore the intersection between student gender and ethnicity. Through this analysis, Sorkhou et al. (2022) identified significantly higher levels of psychological distress among South and East Asian students compared to White students with a further interaction between gender and ethnicity for predicting greater distress. The authors argue that, with correct adoption of outcome measures like the OQ-45, these data show promise for informing targeted or personalised interventions to support student mental health within educational settings.

### Assessment and academia

An important consideration for service providers lies in utilizing measures that effectively capture their impact on academic variables while remaining responsive to student needs. University and college counseling services are increasingly expected to showcase their role in enhancing holistic student aspects, encompassing mental health, academic distress, institutional belongingness, and fostering independent learning (Murray et al., 2016). The impact of counseling on academic domains has been demonstrated through practice-based evidence, highlighting its effectiveness even when academic factors are not the primary purpose of counseling and studies have revealed clinically significant improvements for students despite facing academic challenges (see Biasi et al., 2017; McKenzie et al., 2015).

A recent study by Scruggs et al. (2023) utilized real-world data from two university counseling services employing the CORE-OM in conjunction with standardized questions about the influence of counseling on academic proficiency. These services used the Counselling Impact on Academic Outcomes (CIAO) questionnaire, which is widely used in UK university counseling services and analyses of the CIAO data revealed that counseling reduced students’ self-reported problems affecting their academic ability. While opportunities for enhancement exist in the CIAO’s design and application, it offers a means for universities and colleges to showcase how counseling, as reported by students, mitigates challenges affecting their academic achievements. Collectively, these investigations underscore the value of services incorporating academic data, such as the CIAO, to substantiate their broader contributions to student outcomes.

### Routine outcome monitoring and matching

To maximize the utility of measurement, counseling centers can adopt routine outcome monitoring (ROM) which, in its simplest form, involves gathering regular patient/client data, providing feedback to therapists and clients based on the data, and adjusting counseling or therapy as necessary. This approach significantly enhances collaborative decision-making, treatment understanding, and the therapeutic alliance by involving patients in outcome monitoring and facilitating feedback integration during therapy sessions (De Jong et al., 2021). Recent evidence demonstrates that successful ROM implementation can be cost-effective and, while yielding effects that might traditionally be viewed as small, importantly these are *additional* to those of standard psychological therapies, equating to an approximate 8% advantage (Barkham et al., 2023).

Despite the potential benefits of ROM, there is unanimous agreement regarding the challenges of implementation, a situation that may be particularly acute in student settings. For example, Mellor-Clark et al. (2016) emphasize aligning ROM procedures with behavior change strategies and implementation science for effective and sustainable monitoring. They underscore the importance of organizational readiness, proactive problem-solving, resource development, and comprehensive training to ensure high-quality data collection.

Data-informed approaches in psychological therapies more generally have also been used to improve treatment outcomes by matching clients with therapists (Constantino et al., 2021; Delgadillo et al., 2020). These studies identified specific therapists that were more effective for particular patient subgroups, suggesting that strategic allocation of patients based on profiles could enhance treatment outcomes. These findings showcase the potential of data-informed methods to optimize therapist-patient matching and emphasize the value of early risk identification for enhanced psychological care. However, matching students to practitioners may not be a viable option in that the pressures on time and staff availability may mean that it is more important to be seen sooner rather than wait for a specific therapist. There may also be a debate about preferences, but these always need to be considered in the context of the presenting issues.

### Actionable data and data-informed services

The methods and applications used in these studies can enhance university and college counseling services by leveraging technology, data science, and empirical approaches to improve treatment outcomes for students. This includes transforming in-house counseling services into data-informed spaces, enabling improved decision-making, prediction of risks and outcomes, and identification of mental health subtypes among students. By adopting data-informed approaches, counseling services can gain insights into student needs and preferences, while providing tailored interventions to ensure effective support. These approaches are particularly promising as a way to optimise student outcomes in settings where therapy is typically very short term, with median session numbers falling around 2–4 sessions and noticeably dropping off after 10 sessions in the UK (see Broglia et al., 2019). The use of data-informed decision tools and predictive models has the potential to enhance treatment efficiency, reduce dropout, and identify student groups with varying responses to interventions. These tools can also facilitate decisions about resource allocation, proactive intervention, and support for complex cases based on individual student characteristics.

Regardless of the actual measure used, there is a key point to distinguish between data for data’s sake versus actionable data. This issue relates to both the *function* of the data (why collect it) and to its *form* (how it is presented). In terms of function, there needs to be an intrinsic understanding and relevance as to why data is being collected and the ability to state the reason when asked by students. The collection of data should be underpinned by an implicit memorandum of understanding between the student and clinic as to why the data is being requested and for what purpose.

In terms of form, there needs to be agreement as to how outcomes are reported, and how the subsequent data are used, that might provide higher-level insights rather than measure-specific ones. Such insights also need to be clinically relevant and used in a way to inform decisions that benefit current and future clients. De Beurs et al. (2022) have proposed three specific actions to improve the collection, comparison, and utilization of data: 1) use common metrics, such as T scores and percentile ranks derived from raw scores; 2) develop crosswalk tables and formulas to aid the conversion of scores into common metrics; and 3) collecting normative data from community samples and conducting international validation studies. A further approach is to adopt Jacobson and Truax’s (1991) criteria for determining reliable/clinical improvement (see also Evans et al., 1998). This approach has moved outcomes reporting beyond significance reporting but is premised on the psychometric properties of each outcome measure, making good reliability a premium hallmark. Beyond the specifics, while the criteria of reliable and clinically significant improvement comprise the most stringent and exacting threshold, it is only applicable to clients who are scoring at or above clinical caseness at the time of presentation. Hence, to be inclusive of all students referring to a clinic, the adoption of *reliable improvement* is likely to be a more inclusive and appropriate criterion.

## Theme 3: involving student and stakeholder perspectives

The third theme takes account of the context of a university with a focus on the culture or climate of the organization as reflected in the involvement of students and other stakeholders. Whilst data is a crucial component, it requires all members of the organization to be open to learning from data that is collected as a natural part of the process. Clients and clinicians should also be at the heart of consent procedures to ensure transparency over the collection and utility of their data.

### Towards a whole university (and college)

Understanding and enhancing mental health support within university settings requires a comprehensive approach that involves students, academic staff, and practitioner perspectives. Co-creation panel discussions with students indicate that their perspectives and proposals for improving mental health support at universities include enhancing the accessibility and effectiveness of services to respond to diverse student groups. Notably, recommendations focused on improving existing services and providing evidence of their effectiveness to inform help-seeking decisions rather than introducing new ones and to ensure that such services are integrated within the broader structural context (Priestley et al., 2022). Similar co-creation research highlights the value students place in enhancing interpersonal and social relationships to improve their mental health and such evidence emphasizes the significance of inclusive social interactions with academic staff, peers, and the local community (see Priestley et al., 2022).

One novel and effective method that has been shown to meaningfully involve students in their mental health support is adopting a grassroots strategy to build community-driven mental health initiatives within educational institutions. An early grassroots initiative aimed to address support gaps for grieving students by establishing a national student-led organisation with peer support groups embedded across campuses. The peer-support programme brought together students across faculties to alleviate isolation and complement university services (Fajgenbaum et al., 2012). A more recent example from Doherty et al. (2021) showcases effective collaboration between grassroots lived experience groups and social work academics to address systemic advocacy and challenge entrenched power imbalances across educational institutions. Their work involved activities to foster trust-building and meaningful engagement between grassroots groups, academics, and services. The overarching enablers from these grassroots examples include developing clear objectives, deep listening, and genuine partnerships to bring about systemic changes within the mental health support framework.

Brewster et al. (2022) highlight the relationship between staff and student well-being in university settings and argue for a proactive and cohesive approach to embed cultural and structural changes throughout the institution to promote positive well-being outcomes for the entire university community. They suggest that such a strategy relies on considering the interconnectivity of staff and student well-being and a commitment to update institutional policies, training, and culture accordingly to consider the role of workplace culture in supporting well-being and a healthy academic environment. Students advocate mentally healthy university environments with accessible support services, prioritizing individual needs (Priestley et al., 2022). Olaniyan and Hayes (2022) explored culturally appropriate support strategies for racial and ethnic minority groups, favoring person-specific services that emphasize cultural relevance and accessibility over rigid protocols. They highlight the importance of fostering humanity and trust through reflective processes that acknowledge cultural differences. Encouraging further research into intersectional influences on help-seeking attitudes among racial and ethnic minorities, this study calls for universities to adopt adaptable approaches to address mental health disparities.

Similarly, Lukenga et al. (2023) studied female students’ perspectives on using digital technologies for academic stress management, uncovering coping mechanisms and the potential of technology adoption to enhance coping strategies and alleviate academic stress. These studies emphasize engaging diverse student stakeholders in research to enhance mental health and academic support with a focus on cultural relevance and individualized approaches. Student and academic staff views on providing a mentally healthy university environment are relatively consistent. For example, Cage et al. (2021) analysed focus group data from UK universities to enhance our understanding of how to improve transitions in student mental health. Both students and staff championed the adoption of a “whole university” approach to mental health, which involves implementing comprehensive and holistic measures throughout the institution to enhance community mental health, wellbeing, and acknowledge the entirety of the student journey. The authors outline a strategic framework encompassing pre-entry transition support, peer mentoring programs, well-defined roles for student services, and assistance during the transition out of university. Notably, both students and staff highlight the significance of effective expectation management, imparting coping skills to students, and nurturing a sense of belonging. Staff members, in particular, voiced the challenges tied to limited resources and heightened demand for mental health services, underscoring the need for enhanced training and resource allocation.

### Including staff

Staff perspectives play a crucial role in mental health research and in shaping university strategies to provide mentally healthy campuses. Research on staff experiences of supporting student mental health reveal both positives and obstacles. For example, while some staff display moderate confidence in providing emotional support, a noticeable proportion of staff feel unprepared or lack formal training and clarity about their role (Gulliver et al., 2018). Likewise, when staff receive training to improve their depression literacy, they feel more equipped to assist students and make informed decisions about sign-posting (Gulliver et al., 2019). While openness to mental health training is evident among academic staff, research suggests that the majority of staff remain untrained (Margrove et al., 2014). Moreover, while this research is insightful, there is limited research exploring the views of academic staff despite their pivotal role in supporting students and acting as gatekeepers to services and information. There is also a distinct lack of research involving professional staff working in universities and colleges in shaping student mental health services and campus policies. When their views have been captured, they describe pressure from university leaders to “do more for less” and they may work defensively though a fear of “getting it wrong” (see Broglia et al., 2023).

Integrating both student and academic staff perspectives is essential for creating effective and holistic mental health support strategies within university environments. While research has shed light on students’ needs and preferences, understanding the challenges and training gaps faced by academic staff is equally crucial and there is a distinct research gap involving professional staff. These combined studies demonstrate the potential impact of offering training to enhance staff confidence and literacy as well as the necessity of considering their views when designing mental health policies. Moving forward, further research should continue to engage diverse stakeholders, including academic and professional staff, to ensure comprehensive and culturally relevant approaches that foster a mentally healthy university community.

### Avoiding a crisis narrative

Given the concerning rise in student mental health issues, it’s crucial to be mindful of the messages conveyed to students. It’s vital to empower and encourage help-seeking rather than discourage or deflate it. This is also true for communications for academic and clinical staff given rising reports of burnout and “compassion fatigue” (e.g., Van Hoy & Rzeszutek, 2022). Bantjes et al. (2023) suggest avoiding a “crisis narrative” when communicating and responding to such data. Specifically, they advocate a paradigm shift that acknowledges the inherent resilience of students rather than solely perceiving them as vulnerable and in need of clinical treatment. They emphasise the importance of research and interventions that prioritise understanding and supporting student resilience, accompanied by responsible messaging that avoids medicalizing everyday experiences. In adopting a balanced public health approach, Bantjes et al. (2023) call for resources that avoid pathologizing student mental health and offer effective interventions that cater to the diverse needs of university and college students.

Additional evidence supporting the adoption of tailored communications and interventions for student sub-populations comes from a recent longitudinal study conducted in the UK, which examined the mental health and wellbeing of university students during the COVID-19 pandemic (Paton et al., 2023). Among the 4,622 participants, five distinct wellbeing trajectories emerged, comprising two stable, two declining, and one substantially improved trajectory. Notably, risk factors for poorer wellbeing were identified, including identifying as LGBT+, self-declaring a disability, or having a history of mental health conditions.

## Theme 4: mapping transitions, access, and systems for student mental health services

The fourth and final theme considers the role of longitudinal data collection that enables connections to be made between organizations across the lifespan of adolescence and young adulthood. It ensures that there is an evidence-base derived from data that can inform ongoing decisions concerning students and their mental health. In this context, we acknowledge the persistent and deeply rooted challenges concerning systems and infrastructure. Establishing connections between data and systems within the university/college mental health ecosystem is vital for services to effectively access and employ their data. However, this integration comes with its own set of challenges.

### Transitions

The beginning of a student’s period of study at university/college might appear to be a logical starting point. However, along with others, we have argued previously that there needs to be a strategic focus on transitions and student mental health *prior* to university/college (e.g., Barkham et al., 2019; Campbell et al., 2022). The term “student” shouldn’t be confined to university/college but should encompass a specific group within the broader population of young people. Therefore, the perspective on student mental health should be more inclusive, covering the period before university/college and extending to the wider youth population. Studies, like the extensive multinational research by Auerbach et al. (2018) involving 13,984 students, provide evidence indicating that mental health conditions for university students typically begin at 14.2 years old. Therefore, a significant number of already vulnerable young adults enter university, where various potential triggers can negatively impact some students.

In response to this situation, there is an argument to invest in better support for youth whilst still at school so they can be better prepared for the subsequent experience of university/college. Attention has also focused on the potential preventative and facilitative role of wellbeing interventions to foster student mental health. For example, systematic reviews have considered school-based programs in the UK (Mackenzie & Williams, 2018), while Gunawardena et al. (2023) reviewed programs in Australia and concluded there to be no substantial enhancement in wellbeing outcomes from such programs. Therefore, despite the appeal of such programs, questions persist regarding their effectiveness.

One example of an attempt to address mental health early in the youth timespan was the MYRIAD (MY Resilience In ADolescence) project, a large 7-year UK program offering mindfulness training and involving 28,000 11–16 year olds and 650 teachers across 100 schools (Kuyken et al., 2022). The rationale for the project was relatively simple, namely, that mindfulness worked for adults (Hofmann et al., 2010). Unfortunately, the results did not support the provision of mindfulness at the level of individual pupils, with subsequent analyses reporting that 80% of pupils only practiced mindfulness once (Montero-Marin et al., 2023). However, the researchers concluded that it positively impacted staff burnout and the school climate overall. Several important lessons arise from this research. First, it cannot be assumed that what works for adults will transpose to youth; there has to be relevance and a suitable fit to students rather than assume that interventions supported in the adult population will generalize. Second, requiring teachers to deliver mindfulness proved unsuccessful, with a limited number considered competent (Crane et al., 2020) and raising the important case for recognising that supporting mental health requires specialist training. And third, it may be as important to focus on the organization and its personnel as much as on students themselves given that it is within the organization itself that so many of the potential triggers for negative experiences reside. Consequently, a prudent and research-driven approach is advisable when considering the adoption and assessment of student wellbeing interventions.

### Connections and longitudinal cohort studies

A bridge to address the potential disconnect between schools and universities and the associated transition would be to establish a national research strategy premised on the implementation of longitudinal cohort studies that provide trend data across the continuous time span of youth. But crucially, such data needs to relate proximally to the specific student profile of an institution. Many surveys of university students draw on samples from single institutions and single subjects (e.g., psychology) that make either generalizations or targeted information beyond those single institutions vulnerable.

By contrast, national and strategic planning and investment in representative sampling of youth from early adolescence would yield a rich resource of actionable data, which is, in itself, a key component for universities and colleges as learning health systems. This is a primary principle upon which the recommendations in this article are based, namely, the collection of reliable, relevant, and actionable data that is embedded within the university/college mental health support services. Such insights are essential to counteract various pressures faced by universities/colleges. Firstly, to address low-quality studies producing unsupported conclusions that trigger immediate responses. Secondly, to manage media pressure following student suicides. Lastly, to reassure parents and guardians by ensuring access to pertinent information guiding their decisions for their students.

### Access and mapping in student mental health services

Focusing specifically on the use of mental health services at university/college, it is important to assess the effective utilization of such services by university students and to consider the rise in mental health issues and the corresponding uptake of services within the general student population (Ayón et al., 2022; Lipson et al., 2019). However, there may be a greater demand for mental health support as some students choose not to disclose their conditions and certain services remain underutilized (Abreu et al., 2017). Disparities in access also persist for marginalized groups, including men, minority ethnic groups, and mature students (Lipson et al., 2022; Sagar-Ouriaghli et al., 2020). Mapping student access, system infrastructure, and the policies surrounding mental health services is necessary to understand service use and access complexities.

Several studies have explored the use of mental health services among university and college students and identified key factors that affect access and utilization. In the United States, Lipson et al. (2019) conducted a population-level analysis over a 10-year period and found significant increases in mental health treatment rates, lifetime diagnoses of mental health conditions, and a decrease in stigma. These trends indicate a positive shift in attitudes toward seeking help and increased recognition of mental health issues among students. However, differing trends have emerged since the COVID-19 pandemic. For example, Lee et al. (2021) found that the COVID-19 pandemic had a greater impact on students who were female, academically underperforming, from rural campuses, or low-income backgrounds. Despite these deteriorating trends, the majority of students did not utilize mental health services, indicating either a reluctance to seek help or persistent barriers to accessing support.

Disparities in students accessing mental health services exist between countries and within different student populations. In a recent meta-analysis, Osborn et al. (2022) highlight significant variation in student service utilization and a paucity of studies outside the USA. The authors call for international studies into student access as well as efforts to develop service partnerships to improve student access. Attention is also needed to adapt student care pathways and interventions to respond to their unique challenges as evidence suggests that students receiving routine psychological therapy from national services (e.g., the English National Health Service) do not experience the same level of recovery as the general population (Barnett et al., 2022).

Mapping student access to and transitions through mental health services is a novel approach that offers numerous benefits for higher education institutions. O’Brien et al. (2020) provided insights into student mental health concerns and educational requirements by mapping support pathways and developing tailored referrals and interventions. Using a mixed-methods approach, they developed a customized mental health program incorporating behavior change techniques to encourage help-seeking behaviors among undergraduates. Mapping techniques have also been used to tailor referral pathways and interventions for medical students, to promote early help-seeking and to address concerns about fitness to study. Effective strategies in this context involve collaborative efforts with medical schools and online student platforms to establish trust, deliver clear messaging, customise referral pathways, and ultimately foster a positive culture for seeking help (e.g., Jacob et al., 2020; Shahaf-Oren et al., 2021).

The recognition of mapping student referral pathways to mental health services has significantly impacted diverse service networks worldwide. For example, Vallianatos et al. (2019) transformed student mental health services in a Canadian institution through community mapping, network development, and stakeholder engagement. Ongoing monitoring and evaluation allowed the program to adapt to students’ specific needs, facilitating rapid access to services, early case identification, proactive follow-up, and active involvement of students and their families. Similar strategies have been used to develop a centralized student mental health network across Canada to improve mental health literacy, coordinate services, and promote early access to support (see Ecclestone et al., 2023).

Coordinated care pathways are also being developed in the UK. For example, 2023 explored strategies for developing service partnerships between the education and public health sectors. Their evaluation included five student mental health hubs that aimed to foster collaborations between universities and the National Health Service (NHS). Mapping students’ journeys and service data flow revealed essential factors for partnership working, including developing shared language and communication, joint risk management procedures, and clarifying staff roles. Implementing these strategies to address increasing demands has the potential to enhance service access and quality. Efforts have also been made not only to foster partnerships between services but also to unite researchers, practitioners, and students through the establishment of research networks aimed at enhancing student mental health (e.g., Student Mental Health Research Network (SMaRteN), see https://www.smarten.org.uk/; and Inlight Network, see https://smhr.utoronto.ca/).

Identifying gaps within data flow and decision-making processes informs the development of service infrastructure and partnerships with external providers. Moreover, by adopting collaborative approaches and providing targeted support for specific student groups, universities can enhance access to mental health services while addressing stigma and promoting trust and confidentiality for all students.

### Summary from themes

Overall, we have advocated a comprehensive approach, informed by international research and practitioner observations, to address students’ mental health needs. This includes adopting a public mental health perspective comprising professional counseling, evidence-based practices, and interventions that address social determinants of well-being and student sub-groups. Task-sharing models with peer-to-peer support, early identification of at-risk students, pre-university preparations, and promoting mental health literacy contribute to a supportive education environment (Bantjes et al., 2023; Campbell et al., 2022). Meaningful stakeholder involvement and links with grassroots groups are central to adapting support services, policies, and communication to meet the needs of under-represented and marginalized students (Cao et al., 2021).

### Recommendations towards learning health systems

The four themes outlined in this article provide key components for universities and colleges adopting the principles and procedures associated with learning health systems. To aid this process, in this final section, we present recommendations derived from the four themes (see Table 1).

1. *Prioritize high-quality and actionable data*: Incorporate ROM data into clinical practice and treatment planning. Use feedback to inform interventions, track progress, and identify areas for improvement, empowering both students and mental health professionals. This data-informed approach fosters collaboration and enhances treatment outcomes. The collection of high-quality, robust, and actionable data is critical for the transformation into learning health systems, enabling real-time, informed decision-making with user-controlled data utilization.
2. *Standardize recovery criteria and measurement comparisons*: Standardize recovery criteria for consistent outcomes across services and sectors to support collaborations between practitioners and combining research evidence. Promote strategies to compare measures across diverse student groups and settings such as adopting common metrics and creating meaningful classifications. These strategies will enhance treatment planning and reduce measurement burden.
3. *Leverage embedded data from services*: To enhance data collection efficiency, colleges and universities can leverage embedded data within existing systems to provide seamless monitoring and understanding of student mental health.
4. *Facilitate learning health systems*: Transform colleges and universities into learning health systems that continuously learn from data, empower decision-makers, improve student experiences and outcomes. This transformation ensures services effectively utilize data for better service delivery and student support while implementing data-informed approaches will lead to better service access, quality, and outcomes for students.
5. *Promote choice and flexibility for selecting measures*: Enable services to choose measures that are strategic but aligned with their specific goals and capabilities. Encourage the adoption of a battery of brief domain-specific or multi-domain measures to ensure comprehensive data collection. Additionally, consider the acceptability of measures among students and service users to capture cultural diversity needs accurately. This proactive approach allows for a tailored and inclusive data collection process that supports the overall effectiveness of the measures used.
6. *Enhance assessment and measurement strategies*: Recognize the distinct functions of assessment and outcome measures, understanding their intended uses, such as risk assessment, profiling needs, or determining recovery. Select measurement tools with sensitivity to encompass psychological, functioning, and academic aspects, ensuring a holistic view of student well-being.
7. *Promote inclusivity through stakeholder involvement*: Explore gaps in mental health support for under-represented and marginalized student groups by involving stakeholders in the evaluation and adaptation of support services and policies. Research the acceptability and validity of measures used with these groups for more inclusive data collection and analysis. Collaborate with academic and professional staff to actively participate in research and the development of services to cultivate campuses that prioritize mental health and wellbeing. Engage staff and practitioners as co-creators in shaping university policies, fostering a holistic approach to mental health within the entire academic community.
8. *Develop partnerships and data linkage strategies*: Facilitate partnership development between public healthcare and education sectors to enable ethical data integration and offer a comprehensive and holistic view of student mental health. Prioritize transparency and collaboration in documenting data collection procedures to promote data comparability and reproducibility, allowing identification of trends and best practices.
9. *Collect data pre- and during student transitions and service access*: To gain valuable insights, collect data on student mental health during transitions from pre-university to university. Map student journeys from accessing services to ongoing experiences for a holistic view and inform targeted interventions to support seamless transitions.
10. *Establish longitudinal cohort studies*: Develop national and international cohort studies to produce longitudinal mental health and well-being insights in students. This comprehensive approach deepens understanding of trends and informs evidence-based decision-making.

**Table 1.** Summary recommendations to aid the collection of actionable data to improve student mental health

Themes		Recommendations
Learning from global insights on university students’ mental health	1.	Prioritize high-quality and actionable data
	2.	Standardize recovery criteria and measurement comparison
	3.	Leverage embedded data from services
	4.	Facilitate principles and practices of learning health systems
Measuring, monitoring, and managing data	5.	Promote choice and flexibility for selecting measures
	6.	Enhance assessment and measurement strategies
Involving student and stakeholder perspectives	7.	Promote inclusivity through stakeholder involvement
	8.	Develop partnerships and data linkage strategies
Mapping transitions, access, and systems for student mental health services	9.	Collect data during student transitions and service access
	10.	Establish longitudinal cohort studies

The recommendations in this article are derived from interdisciplinary evidence to highlight examples of good practices for improving the collection and utilization of student mental health data. They broadly endorse a comprehensive, public health-oriented approach, covering areas such as overcoming treatment barriers, coordinating services, and improving measurement practices. By implementing these recommendations, we argue that educational institutions can foster supportive environments that promote student mental health by aspiring to be a learning health system that thereby protects academic success. Importantly, while learning health systems can be implemented using a “top-down” model, that is, informed by national registries, they can also be instigated by adopting a “bottom-up” model within single institutions and then building across other organizations with a “think globally, act locally” mantra (Smoyer et al., 2016).

## Conclusion

In becoming learning health systems, universities and services must prioritize the quality, robustness, and actionability of their data. Embracing these principles creates a dynamic ecosystem that leverages data for continuous improvement and informed decision-making, advancing student well-being and achieving learning health system goals. Through comprehensive data capture and implementation of robust data collection methods, universities and colleges can create an environment where data becomes a powerful tool in enhancing health outcomes and student experiences.
